# Exploring pre-diagnosis hospital contacts in women with endometriosis using ICD-10: a Danish case–control study

**DOI:** 10.1093/humrep/deae273

**Published:** 2024-12-20

**Authors:** Anna Melgaard, Claus Høstrup Vestergaard, Ulrik Schiøler Kesmodel, Bettina Wulff Risør, Axel Forman, Krina T Zondervan, Mintu Nath, Dolapo Ayansina, Philippa T K Saunders, Andrew W Horne, Lucky Saraswat, Dorte Rytter

**Affiliations:** Research Unit of Epidemiology, Department of Public Health, Aarhus University, Aarhus C, Denmark; Research Unit for General Practice, Department of Public Health, Aarhus University, Aarhus C, Denmark; Department of Obstetrics and Gynecology, Aalborg University Hospital, Aalborg, Denmark; Central Denmark Region, DEFACTUM, Aarhus N, Denmark; Department of Clinical Medicine, Danish Centre for Health Services Research, Aalborg University, Gistrup, Denmark; Department of Obstetrics and Gynecology, Aarhus University Hospital, Aarhus N, Denmark; Nuffield Department of Women’s and Reproductive Health, Oxford Endometriosis CaRe Centre, University of Oxford, John Radcliffe Hospital, Oxford, UK; Institute of Applied Health Sciences, University of Aberdeen, Aberdeen, UK; Institute of Applied Health Sciences, University of Aberdeen, Aberdeen, UK; EXPPECT Edinburgh, Centre for Reproductive Health, Institute of Regeneration and Repair, University of Edinburgh, Edinburgh, UK; EXPPECT Edinburgh, Centre for Reproductive Health, Institute of Regeneration and Repair, University of Edinburgh, Edinburgh, UK; University of Aberdeen, Aberdeen, UK; Research Unit of Epidemiology, Department of Public Health, Aarhus University, Aarhus C, Denmark

**Keywords:** endometriosis, diagnostic delay, health care utilization, women’s health, case–control study, comorbidity

## Abstract

**STUDY QUESTION:**

How does pre-diagnosis use of hospital care differentiate between women later diagnosed with endometriosis and age-matched controls without a diagnosis?

**SUMMARY ANSWER:**

Women with hospital-diagnosed endometriosis had more frequent hospital contacts in the 10 years leading up to the diagnosis compared to women without a diagnosis of endometriosis, and the contacts were related to registered diagnoses in nearly all of the included ICD-10 chapters for the entire period.

**WHAT IS KNOWN ALREADY:**

Only a few studies have investigated the utilization of health care among women with endometriosis in the time before diagnosis, but current research shows that women with endometriosis have a higher utilization compared to women without diagnosed endometriosis. To our knowledge, no study has investigated the type of contact related to the higher utilization by using the ICD-10 diagnoses registered to the hospital contact.

**STUDY DESIGN, SIZE, DURATION:**

This study was conducted as a national Danish registry-based case–control study of 129 696 women. Cases were women with a first-time hospital-based diagnosis of endometriosis between 1 January 2000 and 31 December 2017.

**PARTICIPANTS/MATERIALS, SETTING, METHODS:**

Using density sampling, we identified 21 616 cases. Each case was matched on age at the date of diagnosis (index date) to five women without hospital-diagnosed endometriosis (n = 108 080) at the time of matching. The utilization and registered ICD-10 diagnoses related to the hospital contact were included for the 10 years before the index date.

**MAIN RESULTS AND THE ROLE OF CHANCE:**

The probability of having a high number of hospital contacts (six or more) was more common among women with endometriosis (68.6%) compared to women without endometriosis (55.7%) In general, women without endometriosis were more likely to have fewer than six contacts. The diagnoses registered to the contact among cases were related to a greater variety of ICD-10 chapters when compared to controls with the same number of contacts. For nearly all of the included ICD-10 chapters, women with endometriosis were more likely to have a diagnosis over the entire period compared to controls, with the only exception being in the chapter related to pregnancy.

**LIMITATIONS, REASONS FOR CAUTION:**

Our results are only applicable for women with hospital-based diagnosed endometriosis since we were not able to include women diagnosed at the general practitioner or private gynecologists. We were not able to make a causal interpretation, as we do not have information on the onset of symptoms of the included diseases. The association may be overestimated due to detection bias. However, a sensitivity analysis only changed the results slightly, indicating a low risk of this bias.

**WIDER IMPLICATIONS OF THE FINDINGS:**

This study is in accordance with previous studies on the subject, indicating that the utilization of health care prior to endometriosis is not necessarily restricted to endometriosis-related symptoms and that endometriosis can be associated with many other diseases. Future studies may explore hospital contacts and causes/diagnoses following the endometriosis diagnosis to further shed light on whether our results are due to a pattern of multiple pathologies or rather an expression of misdiagnoses among women with endometriosis before diagnosis.

**STUDY FUNDING/COMPETING INTEREST(S):**

This study is supported by grants from the project *Finding Endometriosis using Machine Learning* (FEMaLe/101017562), which has received funding from The European Union’s Horizon 2020 research and innovation program and Helsefonden (21-B-0141). A.W.H. received grant funding from NIHR, CSO, Roche Diagnostics, and Wellbeing of Women. A.W.H.’s institution received consultation fees from Theramex, Joii, Gesynta, and Gedeon Richter. A.W.H.’s institution received honoraria for lectures from Theramex and Gedeon Richter. A.W.H. is listed as co-inventor on a patent application (UK Patent App No. 2217921.2, International Patent App No. PCT/GB2023/053076). P.T.K.S.’s institution (University of Edinburgh) received consultation fees from Gesynta Pharma AB and BenevolentAI Bio Ltd. P.T.K.S’s institution (University of Edinburgh) declares a patent application (UK Patent Application No. 2310300.5. Androgens in diagnostic strategies for endometriosis). P.T.K.S. is a treasurer of the World Endometriosis Society, Fellowship in the Royal Society of Edinburgh, and a Scientific Advisor of the Royal College of Obstetrics and Gynaecology.

**TRIAL REGISTRATION NUMBER:**

N/A.

## Introduction

Endometriosis is a gynecological disease estimated to affect 10% of women of childbearing age (Shafrir *et al.*, 2018). The most common symptoms are painful periods, chronic pain, and infertility. It is a complex condition to diagnose, due to a symptomatic overlap with other gynecological and gastrointestinal diseases, and a requirement of imaging or surgical visualization, most commonly via laparoscopy, to have a definite diagnosis (Zondervan *et al.*, 2020; Becker *et al.*, 2022). Because of its complex nature, endometriosis is associated with under-diagnosis and a long diagnostic delay. Women with endometriosis often describe the diagnostic process as protracted, including repeated consultations with medical professionals, referral to wrong specialists, and misdiagnoses (Pettersson and Bertero, 2020; Surrey *et al.*, 2020; van der Zanden *et al.*, 2021). Additionally, the time before a definite endometriosis diagnosis is made can also include symptom management based on a suspected diagnosis.

Endometriosis is found to be associated with co-morbidities like irritable bowel syndrome, depression/anxiety, certain types of cancer, asthma, and some autoimmune, cardiovascular, and atopic diseases (Kvaskoff *et al.*, 2015, 2021; Surrey *et al.*, 2018).

Previous studies have found that women with endometriosis have higher utilization of health care before receiving the diagnosis compared to women without endometriosis (Ballard *et al.*, 2008; Surrey *et al.*, 2020; Melgaard *et al.*, 2023). However, the current knowledge is still sparse on why they have a higher number of health care contacts.

The symptomatic overlap of endometriosis with other conditions and/or potential misdiagnoses may account for the higher pre-diagnosis utilization of health care in women with endometriosis. However, there remains limited knowledge of the hospital contacts before the endometriosis diagnosis. Thus, the aim of this study was to describe the registered diagnoses based on ICD-10 codes for women with endometriosis who attended the hospital in the 10 years before their diagnosis, and to compare this to a group of age-matched women without diagnosed endometriosis.

## Materials and methods

We conducted a national Danish case–control study. The study population has been described in detail elsewhere (Melgaard *et al.*, 2023). Briefly, we identified the source population through the Danish registers using the unique civil personal registration number (CPR number) that all Danish citizens are assigned. Women entered the source population when the study period started on 1 January 2000, on their 15th birthday, or when they had lived in Denmark for at least 10 consecutive years, whichever came last. They left the source population when the study period ended on 31 December 2017, on their 55th birthday, date of emigration from Denmark, death, or date of receiving a first-time hospital-based diagnosis of endometriosis. All women diagnosed with endometriosis were identified as cases (hereafter referred to as cases or women with endometriosis) at the date of diagnosis, and each case was age-matched with five controls (hereby referred to as controls or women without endometriosis), who were women without a hospital-based diagnosis of endometriosis at the time of matching. The date of diagnosis was the index date. All women who had received a diagnosis of endometriosis before entering the source population were identified and excluded beforehand.

### Endometriosis diagnosis

Information on endometriosis diagnosis was obtained from the Danish National Patient Registry which contains information on all public hospital contacts and diagnoses from 1977 and for all private hospital contacts and diagnoses from 2002 (Schmidt *et al.*, 2015). The date of first hospital contact where the main reason for contact was endometriosis (registered as an A-diagnosis) or because endometriosis was present, but not the main reason for contact (registered as a B-diagnosis) was included as the date of diagnosis. We included the following diagnoses to identify endometriosis: ICD-8 codes 62530 and 62532–62539 from 1977 to 1994 and ICD-10 codes DN801–809 from 1994 onward. Women were diagnosed either with imagining or surgery, and 58% of the cases had a histologically confirmed diagnosis.

### Hospital contacts and ICD-10 diagnoses

We included information on the number and date of all public and private hospital contacts, including in-patient admissions, out-patient visits, and emergency visits with somatic and psychiatric departments in the 10 years preceding the index date. Information on contacts with psychiatric departments was only available in the registry from 1995 and onward. For private hospitals, the registry only holds information from 2002 and onward, but the majority of patients are managed in the public health care system (98.75% of all contacts in 2017) (Holstein, 2019).

We identified the main reason for each hospital contact (A-diagnoses), and categorized them according to the chapters in the ICD-10 classification system (World Health Organization, 2018). From 1990 to 1994 when ICD-8 was used, we categorized the ICD-8 diagnoses in accordance with ICD-10 chapters using instructions from Statistics Denmark. We excluded chapter XVI ‘certain conditions originating in the perinatal period’ as this was irrelevant to the study population.

### Covariates

We used the CPR numbers to link data from the included Danish Registers. Information on date of birth, region of residence, ethnicity (Danish, immigrant, or descendant of an immigrant) household composition (single, couples, or other), and migration in and out of Denmark was obtained from the Danish Civil Registration System (Erlangsen and Fedyszyn, 2015).

We included information on the highest educational level completed and labor market affiliations as proxies for socioeconomic status. Education information (primary, upper secondary, short cycle tertiary or BA, master or equivalent, or PhD) and labor market affiliation (self-employed/executive, employed, on social benefits, student, or other) was obtained from Statistics Denmark’s register on education and family income. For labor market affiliation, we included the highest status in the household. The information on labor market affiliation was first recorded in the registers from 1994 onward. Therefore, we used information from 1994 as a replacement for the missing years (1990–1993) with an acceptance of a slight inaccuracy in exchange for including the data. For women aged 15–25 years, we used information about the educational level of the parents, under the assumption that the parent’s status had a higher impact on the woman’s health care-seeking behavior and health status when they are young.

### Statistical analysis

The number of contacts (ranging from ‘≥1’ to ‘≥6’) was tabulated for cases and controls. This was done for the entire period of 10 years, but also for the last year before the index date, 1–5 years before the index date, and 6–10 years before the index date. The prevalence of different numbers of contacts was compared among cases and controls with a prevalence proportion ratio (PPR) and corresponding 95% CI.

To investigate whether the hospital contacts were related to the same or various ICD-10 chapters, the number of different chapters registered as the main diagnosis (ranging from ‘1’ to ‘≥6’) corresponding to the number of hospital contacts (ranging from ‘1’ to ‘≥6’) for cases and controls was visualized by a stacked bar graph.

Using conditional logistic regression analysis, we estimated odds ratios (ORs) and corresponding 95% CI of having a main diagnosis in each of the specific ICD-10 chapters among cases compared to controls. As before, this analysis was stratified into the last year leading up to the index date, 1–5 years before the index date and 6–10 years before the index date, to investigate changes over time. The model was adjusted for the included covariates: age as the matching variable, region of residence, educational level, household type, labor market affiliations, and ethnicity.

Since the analysis showed noticeably large ORs for specific ICD-10 chapters, particularly in the year leading up to index date, a *post hoc* analysis was conducted for this year to investigate the subcategories for underlying diagnoses in chapters with an OR larger than 2.0.

By including cases who received their first-time hospital-based diagnosis of endometriosis as a B-diagnosis (where endometriosis was present, but it was not the main reason for that specific hospital contact), we may have oversampled cases with other conditions. To take this into account, we conducted a sensitivity analysis, restricting cases to only include women who had a diagnosis of endometriosis registered as an A-diagnosis and their respective controls. We estimated the adjusted OR and corresponding 95% CI in each of the ICD-10 chapters.

All statistical analyses were performed in Stata 17 (StataCorp. 2021. College Station, TX, USA: StataCorp LLC).

### Ethics

The study was approved by the Danish Data Protection Agency under the Aarhus University comment agreement and Aarhus University j.number 2016-051-000001, sequential number 1242 (Date: 27 September 2018). According to Danish legislation, ethical approval of registry studies is not required.

## Results

The characteristics of the study population are described in detail elsewhere (Melgaard *et al.*, 2023). In short, the population consisted of 21 616 cases who had a first-time hospital-based diagnosis of endometriosis at some point during the study period and 108 080 controls. Among cases, 15 105 (70%) had endometriosis registered as an A-diagnosis when they were identified as a case.

The characteristics of cases and controls are presented in Table 1. The mean age at the index date was 34.6 years (SD 8.9). The only notable differences between cases and controls were in the region of residence, where there was a higher number of cases living in the North Denmark Region, Central Denmark Region, and Region of Southern Denmark compared to controls. Cases were also more likely to be of Danish origin.

**Table 1.** Characteristics of women with hospital-based diagnosed endometriosis (cases) and age-matched women without endometriosis (controls).

Characteristics	Cases (n = 21 616)	Controls (n = 108 080)
Age at matching, mean (SD)	34.6 (8.9)	34.6 (8.9)
Age at matching, n (%)		
15–24	3770 (17.4)	18 861 (17.5)
25–34	7935 (36.7)	39 624 (36.7)
35–44	7086 (32.8)	35 529 (32.9)
45–55	2825 (13.1)	14 066 (13.0)
Year of diagnosis, n (%)		
2000–2002	3430 (15.9)	
2003–2005	3916 (18.1)	
2006–2008	3652 (16.9)	
2009–2011	3725 (17.2)	
2012–2014	3493 (16.2)	
2015–2017	3400 (15.7)	
Region of residence, n (%)		
North Denmark Region	3073 (14.2)	10 981 (10.2)
Central Denmark Region	5390 (24.9)	24 315 (22.6)
Region of Southern Denmark	4603 (21.3)	22 277 (20.7)
Capital Region	5845 (27.0)	34 898 (32.4)
Region Zealand	2699 (12.5)	15 124 (14.1)
Missing	6 (0)	485 (0.4)
Highest educational level, n (%)		
Primary	3902 (18.6)	20 092 (19.3)
Upper secondary	9692 (46.1)	47 172 (45.4)
Short cycle tertiary or BA	5835 (27.8)	27 650 (26.6)
Master or equivalent	1526 (7.3)	8562 (8.2)
PhD	69 (0.3)	488 (0.5)
Missing	592 (2.7)	4116 (3.8)
Household type, n (%)		
Single	4780 (22.1)	24 276 (22.6)
Couples	14 475 (67.0)	70 776 (65.8)
Other	2355 (10.9)	12 543 (11.7)
Missing	6 (0)	485 (0)
Labor market affiliations, n (%)		
Self-employed/executive	1593 (7.4)	8604 (8.0)
Employed	16 843 (77.9)	80 222 (74.6)
Social benefit	1834 (8.5)	10 467 (9.7)
Student	1137 (5.3)	7148 (6.6)
Other*	(≤1)	1153 (1.1)
Missing	(≤1)	486 (0.4)
Ethnicity, n (%)		
Danish	20 350 (94.2)	100 161 (92.7)
Immigrant	1054 (4.9)	6372 (5.9)
Descendant of immigrant*	(≤1)	1461 (1.4)
Missing	(≤1)	86 (0.1)

* Some numbers (and percentages) have been concealed due to few observations in some categories.

This table is similar to the table in Melgaard *et al.* (2023) as the study population is the same.

Over the entire period of 10 years prior to the index date, 97.7% of cases and 91.7% of controls had at least one hospital contact. Table 2 shows that among women with endometriosis, 68.6% had at least six contacts, compared to 55.7% for the controls. The same pattern was present when stratifying the period to 1–5 years and 6–10 years before the index date, however, the largest differences in the number of contacts between cases and controls were found in the last year leading up to the index date. Here, 76.9% of cases had at least one hospital contact, compared to 42.1% for controls. The differences increased with the number of contacts, and 9.8% of cases had six or more contacts in the last year, compared to only 3.5% of the controls.

**Table 2.** The number of hospital contacts for cases and controls for the entire period of 10 years prior to the index date, and stratified into the last year leading up to the index date, 1–5 years before, and 6–10 years before the index date.

Number of hospital contacts	Cases (n = 21 616)	Controls (n = 108 080)	PPR (95% CI)
**All 10 years**			
None	503 (2.3)	8958 (8.3)	0.28 (0.26; 0.31)
Any	21 113 (97.7)	99 122 (91.7)	1.07 (1.06; 1.07)
1	1001 (4.6)	7795 (7.2)	0.64 (0.60; 0.68)
2	1256 (5.8)	8162 (7.6)	0.77 (0.73; 0.82)
3	1378 (6.4)	7871 (7.3)	0.88 (0.83; 0.93)
4	1362 (6.3)	7734 (7.2)	0.88 (0.83; 0.93)
5	1285 (5.9)	7409 (6.9)	0.87 (0.82; 0.92)
6 and above	14 831 (68.6)	60 151 (55.7)	1.23 (1.22; 1.25)
**1 year before the index**			
None	4998 (23.1)	62 557 (57.9)	0.40 (0.39; 0.41)
Any	16 618 (76.9)	45 523 (42.1)	1.83 (1.83; 1.84)
1	5694 (26.3)	18 940 (17.5)	1.50 (1.46; 1.54)
2	3732 (17.3)	10 800 (10.0)	1.73 (1.67; 1.79)
3	2483 (11.5)	6373 (5.9)	1.95 (1.86; 2.04)
4	1567 (7.2)	3610 (3.3)	2.17 (2.05; 2.30)
5	1022 (4.7)	2068 (1.9)	2.47 (2.30; 2.66)
6 and above	2120 (9.8)	3732 (3.5)	2.84 (2.70; 2.99)
**1–5 years before the index**			
None	3872 (17.9)	26 099 (24.1)	0.74 (0.72; 0.76)
Any	17 744 (82.1)	81 981 (75.9)	1.08 (1.07; 1.09)
1	2792 (12.9)	15 291 (14.1)	0.91 (0.88; 0.95)
2	2474 (11.4)	13 121 (12.1)	0.94 (0.91; 0.98)
3	2038 (9.4)	10 950 (10.1)	0.93 (0.89; 0.97)
4	1764 (8.2)	8902 (8.2)	0.99 (0.94; 1.04)
5	1487 (6.9)	7254 (6.7)	1.02 (0.97; 1.08)
6 and above	7189 (33.3)	26 463 (24.5)	1.36 (1.33; 1.39)
**6–10 years before the index**			
None	4622 (21.4)	26 954 (24.9)	0.86 (0.83; 0.88)
Any	16 994 (78.6)	81 126 (75.1)	1.05 (1.04; 1.06)
1	3090 (14.3)	15 730 (14.6)	0.98 (0.95; 1.02)
2	2507 (11.6)	13 194 (12.2)	0.95 (0.91; 0.99)
3	2069 (9.6)	10 749 (9.9)	0.96 (0.92; 1.01)
4	1737 (8.0)	8924 (8.3)	0.97 (0.93; 1.02)
5	1350 (6.2)	6909 (6.4)	0.98 (0.92; 1.03)
6 and above	6241 (28.9)	25 620 (23.7)	1.22 (1.19; 1.25)

Prevalence proportion ratios (PPR) and 95% CIs are also shown. The index date for a case and corresponding age-matched controls is defined as the date of diagnosis of endometriosis of the case.

**Figure 1.**
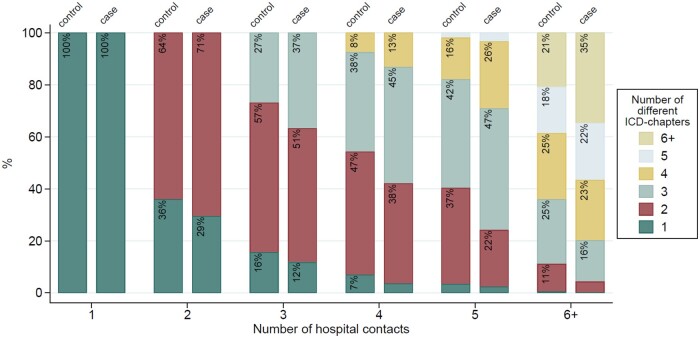
**The percentages of cases and controls by the number of hospital contacts and the number of different ICD-10 chapters in the 10 years before the index date**. The index date for a case and corresponding age-matched controls is defined as the date of diagnosis of endometriosis of the case.

Figure 1 illustrates the distribution of diagnoses in the ICD-10 chapters across the number of hospital contacts for cases and controls in the 10 years preceding the index date. In general, cases had a greater variation of diagnoses from different chapters for the same number of hospital contacts compared to the controls. For those who had two contacts, 29% of cases had contacts related to one chapter, while 36% of controls had contacts within the same chapter. The most notable difference between cases and controls was observed among those with at least six contacts, where 35% of cases had contacts within six or more different chapters compared to only 21% of controls.

**Figure 2.**
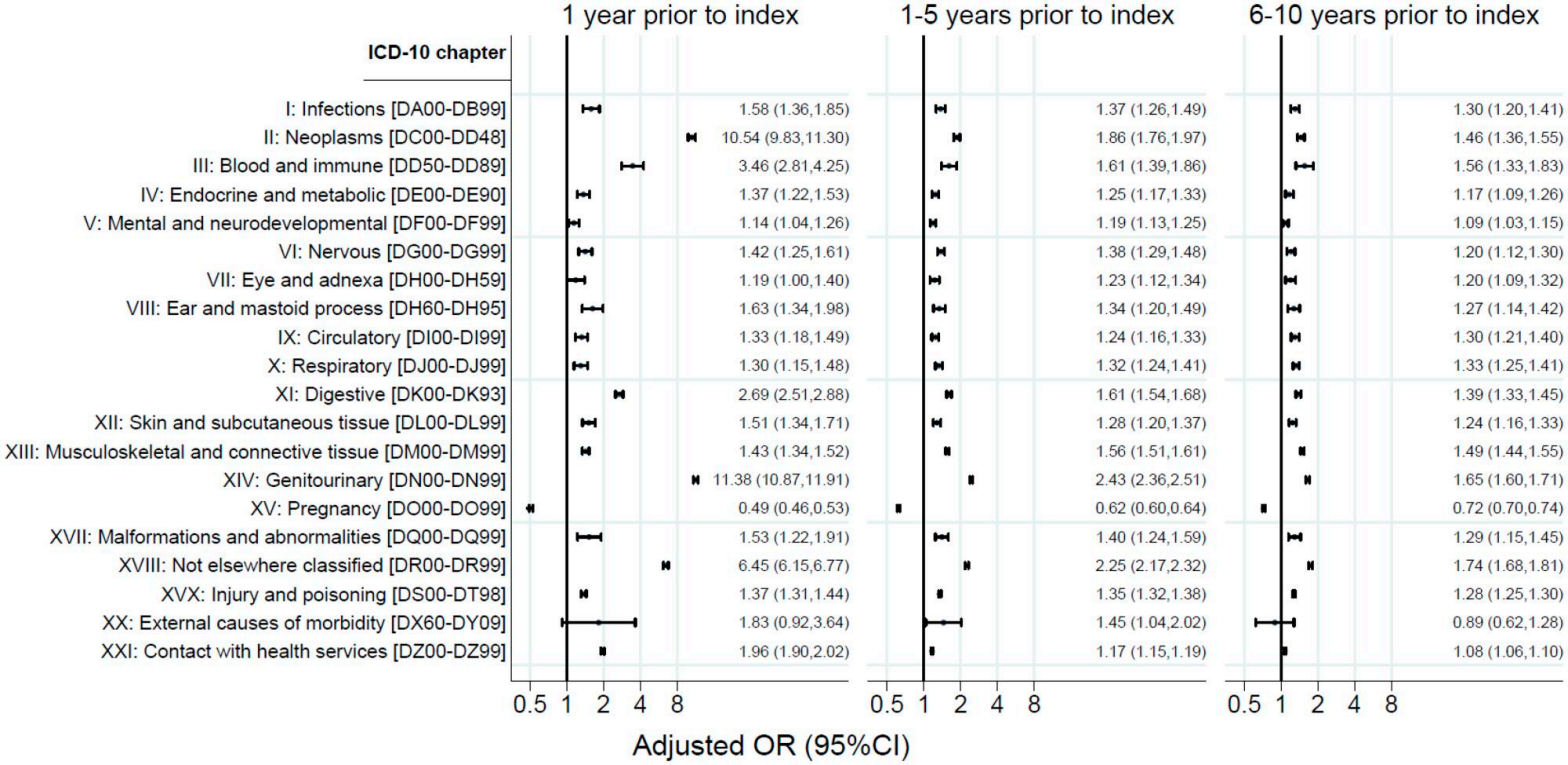
**The adjusted odds ratios and 95% CIs for having a diagnosis in each of the included ICD-10 chapters among cases compared to controls in the 10 years before the index date**. The index date for a case and corresponding age-matched controls is defined as the date of diagnosis of endometriosis of the case.

Figure 2 presents the adjusted OR and corresponding 95% CI for having at least one diagnosis in each of the included ICD-10 chapters comparing cases to controls. This was stratified into the year leading up to the index date, 1–5 years before the index date and 6–10 years before the index date. We observed an association for all applicable chapters and case–control status in each period. For all chapters, cases had a higher probability of having a diagnosis, highlighting a consistent pattern, except in the chapter related to pregnancy where the association was the opposite.

In the period of 6–10 years before the index date, the largest OR was found in the chapter with diseases not elsewhere classified, where cases were 1.74 times more likely to have a diagnosis (95% CI: 1.68; 1.81). In the period of 1–5 years before the index date, the ORs increased slightly for many of the chapters, specifically for the chapters related to neoplasms, diseases of the genitourinary system, and diseases that are not elsewhere classified.

The most profound differences between case and controls were found in the last year leading up to the index date. Here, cases were 10.54 times more likely to have a diagnosis related to neoplasms (95% CI: 9.83; 11.30), 3.46 times more likely to have a diagnosis related to the blood and the immune system (95% CI: 2.81; 4.25), 2.69 times more likely to have a diagnosis of the digestive system (95% CI: 2.51; 2.88), and 11.38 times more likely to have a diagnosis of the genitourinary system (95% CI: 10.87; 11.91). For the chapter related to diseases that are not elsewhere classified, cases were 6.45 times more likely to have a diagnosis registered (95% CI: 6.15; 6.77).

The *post hoc* analysis in Supplementary Table S1 showed, that the strongest association for neoplasms was related to benign neoplasms where cases were 10.63 more likely to have a diagnosis (95% CI: 9.87; 11.44). For diseases in the blood and immune system, the highest OR was related to anemias. For the chapter on diseases of the digestive system, the strongest associations were related to the appendix (OR 3.60 (95% CI: 2.84; 4.56)), hernia (OR 3.60 (95% CI: 2.99; 4.33)), and diseases of the peritoneum (OR 4.58 (95% CI: 2.64; 7.93)). For diseases of the genitourinary system, cases were more likely to have diagnoses related to the kidney and ureter (OR 6.86 (95% CI: 2.91; 16.19)), inflammatory diseases of female pelvic organs (OR 9.76 (95% CI: 8.50; 11.21)), and non-inflammatory disorders of the female genital tract (OR 11.82 (95% CI: 11.27; 12.39)). Finally, for the chapter that contains diagnoses not elsewhere classified, the highest ORs were found for symptoms and signs involving the digestive system and abdomen (OR 9.22 (95% CI: 8.71; 9.78)) and the urinary system (OR 2.53 (95% CI: 2.08; 3.07)).

The results from the sensitivity analysis restricted to cases with endometriosis as an A-diagnosis and their respective controls are shown in Supplementary Fig. S1. Compared to the main analysis, the associations were slightly attenuated, but the pattern was the same. Hence, the largest differences were found in the last year before the index, where the OR for the chapter related to neoplasms decreased from 10.54 in the main analysis to 8.77 (95% CI: 8.06; 9.55) and for the chapter related to diseases of the genitourinary system where the OR decreased from 11.38 to 9.91 (95% CI: 9.38; 10.47).

## Discussion

This Danish nationwide case–control study provides valuable insights into the hospital contacts of women later diagnosed with endometriosis during the 10 years leading up to their diagnosis. Our findings highlight that women with endometriosis had increased health care utilization and wide-ranging registered diagnoses during this time frame. Overall, we found that the probability of having a high number of hospital contacts was more common among women later diagnosed with endometriosis compared to the controls in the 10 years preceding the index date, specifically in the final year leading up to the index date. They were also more likely to have contacts with greater variations of the ICD-10 chapters compared to women without diagnosed endometriosis with the same number of contacts. For all of the included ICD-10 chapters, except the chapter related to pregnancy, women with endometriosis were more likely to have diagnoses for all years compared to controls.

In a previous study on the same study population, we reported that women with endometriosis had a generally higher utilization of in-patient admissions, out-patient visits and emergency visits in the 10 years preceding the diagnosis, compared to women without endometriosis (Melgaard *et al.*, 2023). However, the study did not investigate the proportion of frequent users of hospital care among cases and controls. In the current study, we observed that a higher prevalence of cases had frequent hospital contacts (six or more) compared to the controls, whereas the controls were more likely to have fewer than six contacts. The pattern was evident during the period of 1–5 years and 6–10 years preceding the index date with the strongest association in the last year before the index date. This indicates that the result of the previous study is mainly due to the cases who have frequent hospital contacts.

To our knowledge, there is a lack of research investigating health care utilization among women with endometriosis in the time before receiving the diagnosis. Surrey *et al.* (2020) reported the utilization of health care in the 5 years before diagnosis among women with endometriosis accounting for the length of the diagnostic delay (ranging from ≤1 year, 1–3 years, or 3–5 years). They observed that women with a longer diagnostic delay had a higher number of pre-diagnosis health care utilization, compared to women who were diagnosed earlier after symptom onset. Unfortunately, we do not have access to information on symptom onset in this study. Additionally, Surrey *et al.* reported that the higher use of health care among those with a long diagnostic delay applied to both endometriosis-related and all-cause-related contacts. This corresponds to the findings in Fig. 1 in our study, showing that women with endometriosis were more likely to have contacts related to a variety of different ICD-10 chapters and thereby different diseases, compared to controls with the same number of contacts. This could imply more comorbidity in women with endometriosis or it could represent the difficulty in getting a diagnosis of endometriosis and thereby a high incidence of misdiagnoses over the years before endometriosis is confirmed or even considered. This is a known inherent problem of the diagnosis of endometriosis and one of the reasons for the protracted diagnostic process between symptom onset and diagnosis. However, with the results of this study, we are not able to distinguish between the two explanations.

Our initial hypothesis was that the diagnostic delay of endometriosis could lead to higher health care use and that the non-specific and overlapping symptoms related to endometriosis could lead to wrong referrals and/or misdiagnoses in some other ICD-10 chapters not necessarily related to endometriosis. With the design of this study, we were not able to determine whether the contacts were related to other diseases or misdiagnoses related to endometriosis. However, when exploring the risk of having a registered diagnosis in the included chapters 10 years preceding the index, we surprisingly found, that women with endometriosis had a higher risk of having a diagnosis in almost all the included chapters in all three periods. As expected, since endometriosis is related to infertility (Zondervan *et al.*, 2020), we found that cases were less likely to have a diagnosis within the chapter related to pregnancy. Our results correspond with a recent study by McGrath *et al.* (2023), investigating the relationship between endometriosis and comorbidities in the UK Biobank, where they found that endometriosis was epidemiologically associated with 292 different ICD-10 codes.

A recent Finish study investigated the association between endometriosis and non-gynecological diseases using ICD10 codes and found that women with endometriosis were twice as likely to have non-gynecological diagnoses compared to women without diagnosed or self-reported endometriosis (aOR 2.32 (CI: 1.07; 5.02)) (Rossi *et al.*, 2023). Although the results of our study are aligned with the results from the Finish study, the association is stronger in our study. This could be because we include diagnoses from both in-patient, out-patient, and emergency contacts, whereas the Finish study only includes in-patient contacts. Furthermore, they excluded all contacts related to obstetrics and gynecologic morbidity which certainly must weaken the association compared to our study.

We found a strong positive association of cases with genitourinary diseases as expected, as symptoms of endometriosis are found in this chapter, e.g. pain associated with female genital organs, and female infertility (Zondervan *et al.*, 2020).

Our study obtained a strong association between having a diagnosis with endometriosis and the chapter that includes symptoms, signs, and abnormal results of clinical or investigative procedures with no diagnosis that could be classified elsewhere (chapter XVIII). The association was primarily found for symptoms involving the digestive system and abdomen and urinary system, emphasizing a diagnostic process without final results and the challenges in accurately diagnosing endometriosis due to non-specific symptoms of endometriosis.

We found a higher risk of having a diagnosis in the chapter related to digestive diseases, specifically for diseases in the peritoneum, appendix, and hernia. This could either be linked to non-specific endometriosis-related pain that would lead to an earlier suspicion of these diagnoses, coincidental detection of endometriosis during surgery for some of the conditions in the ICD-10 chapter, or more digestive disease comorbidity in women with endometriosis.

In contrast to the expected association between endometriosis and certain cancer forms (Kvaskoff *et al.*, 2021), we observed a disturbingly large OR for diagnosis in the neoplasms chapter, particularly in the last year leading up to the index date. However, the *post hoc* analysis revealed that most of the diagnoses were related to benign neoplasms including leiomyoma of the uterus and benign neoplasms of the ovary. Hence, the high ORs we found could potentially be explained by the coincidental detection of endometriosis during examination or surgery for these conditions. Our results showed an increased risk of having a diagnosis in the chapter related to diseases of the blood and immune system. The association was mostly related to anemias. Previous studies have investigated the association between endometriosis and anemia suggesting that heavy menstrual bleeding and/or side effects of progestins often used to treat endometriosis could potentially lead to clinically relevant anemia (Strowitzki *et al.*, 2015; Moehner *et al.*, 2020). However, neither of the studies were able to detect any association and found that the medical treatment was generally well-tolerated. A recent study found a genetic overlap between endometriosis and heavy menstrual bleeding; however they were not able to comprehensively assess a causal effect due to lack of power of the data (McGrath *et al.*, 2023). The positive findings in our study related to anemias could be caused by coexisting conditions related to endometriosis such as adenomyosis and uterine fibroids, and we cannot reject that it could be caused by a side effect of medical treatment.

While our large sample size may increase the risk of type 1 error, the magnitude of the association and the associations between endometriosis and clinically relevant conditions reinforces the validity of our findings and their clinical significance.

### Strengths and limitations

A strength of our study was the use of the Danish national registers to identify a large study population from the entire general population. Therefore, we had a well-defined source population at risk which allowed for a robust comparison between cases and controls selected from the general population, which reduced the risk of selection bias on study entry.

The data in the Danish registers were recorded with an administrative purpose, hence it is unlikely that the quality of the exposure (ICD-diagnoses) is influenced by the outcome (endometriosis status) in our study. It is a strength, that we identified cases based on ICD-10 diagnoses, and thereby all diagnoses were medically confirmed. It is a limitation that not all of our cases had a surgical diagnosis, however 58% had a histologically verified diagnosis. Since we do not have access to information on women diagnosed by private gynecologists, the results of this study are only applicable to women with hospital-based diagnosed endometriosis.

Even though it is a strength that we were able to adjust for several potential confounders, unmeasured confounding may still influence our results.

The absence of information on the onset of endometriosis symptoms in individual women limited us to evaluate the temporal progression of the disease. However, the primary objective of this study was to provide an exploratory description of women later diagnosed with endometriosis during the time preceding diagnosis, and not to establish causality.

Women with a more frequent health care-seeking behavior have an increased likelihood of being diagnosed with both endometriosis and other diseases, due to a greater interaction with the health care system in general. This could lead to detection bias and be a partial explanation of the association we found in all ICD-10 chapters. However, it is expected that the proportion of such patients and the risk of confounding is limited, since the general practitioners act as a gatekeeper to hospital care.

In our main analysis, there is a possibility of oversampling cases with other conditions, as we included cases where the main reason for contact when receiving the endometriosis diagnosis, was another condition. This could have led to an overestimation of the associations. However, when we restricted the analyses to cases where endometriosis was given as a main diagnosis, similar associations were found, indicating that Berkson’s bias has affected the results only to a limited degree.

Hormonal medical treatment and analgesics are common treatments to manage endometriosis symptoms (Becker *et al.*, 2022). However, the treatments could also potentially work as mediators and lead to side effects or adverse events, such as headache and nausea (for NSAIDs) (Marjoribanks *et al.*, 2015) and a slightly increased risk of thromboembolic episodes and irregular bleeding (for oral contraceptives) (Brown *et al.*, 2018). This could increase the risk of disease outcomes in this study.

## Conclusion

We observed that women later diagnosed with endometriosis, compared to women without diagnosed endometriosis, had a proportionately higher frequency of hospital contacts and a wider range of diagnoses during the 10-year period preceding the diagnosis. We also found that, overall, women with endometriosis were more likely to have registered diagnoses in almost all of the included ICD-10 chapters in the entire 10-year period. To the best of our knowledge, no previous study has extensively investigated the contact patterns and the registered hospital diagnoses during the 10-year period preceding endometriosis, while also comparing it with the women from the general population without diagnosed endometriosis. Future studies could explore hospital contacts and diagnoses both before and following endometriosis diagnosis and further investigate whether the variety of many diagnoses as seen in this study is mostly related to misdiagnoses before confirmed endometriosis or whether they are in fact comorbidities related to endometriosis.

## Supplementary Material

deae273_Supplementary_Figure_S1

deae273_Supplementary_Table_S1

## Data Availability

Due to restrictions related to Danish law and protecting patient privacy, the combined set of data as used in this article can only be made available through a trusted third party, Statistics Denmark. This state organization holds the data used for this study. University-based Danish scientific organizations can be authorized to work with data within Statistics Denmark and such organizations can provide access to individual scientists inside and outside of Denmark. Requests for data may be sent to Statistics Denmark.
