# Supplementary material for: Exploring pre-diagnosis hospital contacts in women with endometriosis using ICD-10: a Danish case–control study
Source: Hum Reprod. 2024 Dec 20;40(2):280–8. doi: 10.1093/humrep/deae273 (PMC11788223; doi:10.1093/humrep/deae273)
Supplement: deae273_Supplementary_Figure_S1 [file deae273_supplementary_figure_s1.pdf]

Human Reproduction, 2024, 1–1
https://doi.org/10.1093/humrep/deae273
Supplementary Data

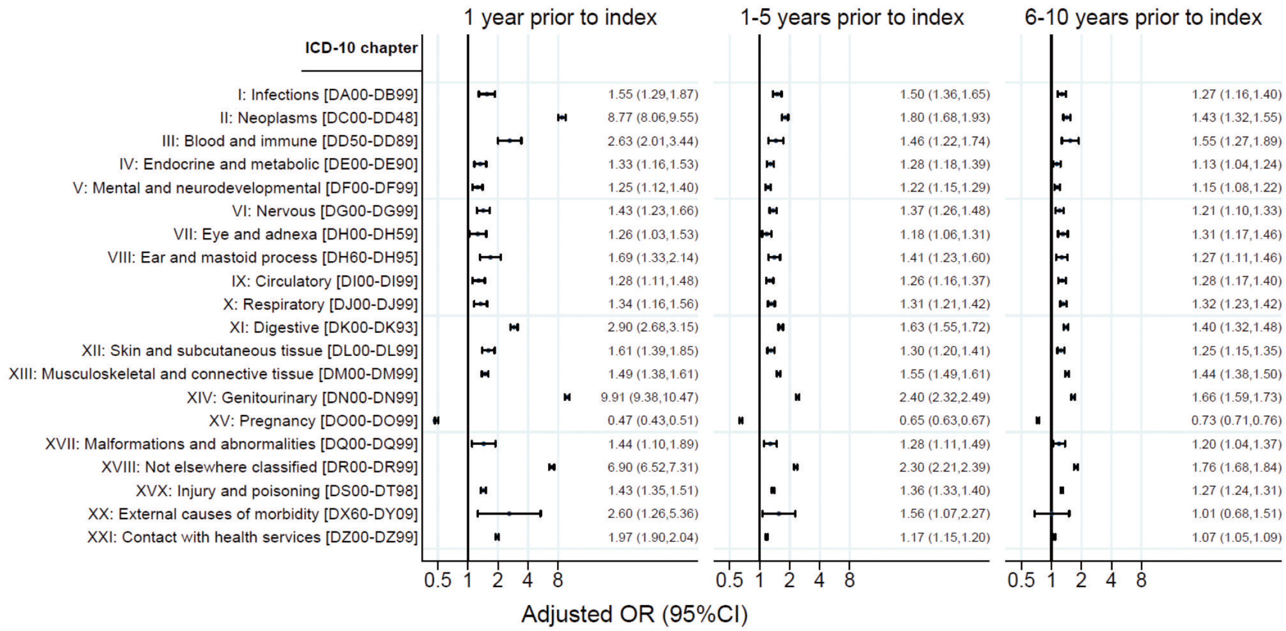

Supplementary Figure S1. Sensitivity analysis of the adjusted ORs and 95% CIs for having a diagnosis in each of the included ICD-10 chapters among cases with endometriosis registered as an A-diagnosis compared to their respective controls in the 10 years before the index date. The index date for a case and corresponding age-matched controls is defined as the date of diagnosis of endometriosis of the case.[1] OR, odds ratio; CI, confidence interval. [1]Stratified into the last year leading up to the index, 1–5 years before, and 6–10 years before the index date. Adjusted for age (matching variable), the region of residence, educational level, household type, labor market affiliations, and ethnicity.
