# Supplementary material for: Exploring pre-diagnosis hospital contacts in women with endometriosis using ICD-10: a Danish case–control study
Source: Hum Reprod. 2024 Dec 20;40(2):280–8. doi: 10.1093/humrep/deae273 (PMC11788223; doi:10.1093/humrep/deae273)
Supplement: deae273_Supplementary_Table_S1 [file deae273_supplementary_table_s1.pdf]

OXFORD human reproduction

Human Reproduction, 2024, 1–1
https://doi.org/10.1093/humrep/deae273
Supplementary Data

Supplementary Table S1. The adjusted odds ratios and 95% CIs for having a diagnosis in subcategories of specific ICD-10 chapters in the last year leading up to the index date.

ICD-chapters and subchapters	OR (95% CI)
**II: Neoplasms (DC00–DD48)**	
DC00–DC97: Malignant neoplasms	1.14 (0.97; 1.35)
DD00–DD09: *In situ* neoplasms	1.51 (1.07; 2.14)
DD10–DD36: Benign neoplasms	10.63 (9.87; 11.44)
DD37–DD48: Neoplasms of uncertain or unknown behavior	4.68 (2.79; 7.86)
**III: Diseases of the blood and blood-forming organs and certain disorders involving the immune mechanism (DD50–DD89)**	
DD50–DD53: Nutritional anemias	3.95 (2.72; 5.73)
DD55–DD59: Hemolytic anemias	6.05 (1.78; 20.48)
DD60–DD64: Aplastic and other anemias	3.84 (2.56; 5.74)
DD65–DD69: Coagulation defects, purpura, and other hemorrhagic conditions	1.28 (0.83; 1.96)
DD70–DD77: Other diseases of blood and blood-forming organs	0.30 (0.05; 1.61)
DD80–DD89: Certain disorders involving the immune mechanism	1.38 (0.82; 2.33)
**XI: Diseases of the digestive system (DK00–DK93)**	
DK00–DK14: Diseases of oral cavity, salivary glands, and jaws	0.70 (0.55; 0.89)
DK20–DK31: Diseases of esophagus, stomach, and duodenum	1.48 (1.22; 1.79)
DK35–DK38: Diseases of appendix	3.60 (2.84; 4.56)
DK40–DK46: Hernia	3.60 (2.99; 4.33)
DK50–DK52: Noninfective enteritis and colitis	1.33 (1.12; 1.58)
DK55–DK64: Other diseases of intestines	2.04 (1.82; 2.29)
DK65–DK67: Diseases of peritoneum	4.58 (2.64; 7.93)
DK70–DK77: Diseases of liver	0.56 (0.29; 1.10)
DK80–DK87: Disorders of gallbladder, biliary tract, and pancreas	0.82 (0.70; 0.97)
DK90–DK93: Other diseases of the digestive system	1.51 (0.98; 2.33)
**XIV: Diseases of the genitourinary system (DN00–DN99)**	
DN00–DN08: Glomerular diseases	1.61 (0.70; 3.73)
DN10–DN16: Renal tubulo-interstitial diseases	4.04 (3.12; 5.24)
DN17–DN19: Renal failure	0.92 (0.55; 1.53)
DN20–DN23: Urolithiasis	1.70 (1.33; 2.17)
DN25–DN29: Other disorders of kidney and ureter	6.86 (2.91; 16.19)
DN30–DN39: Other diseases of urinary system	2.47 (2.14; 2.85)
DN60–DN64: Disorders of breast	1.12 (0.96; 1.32)
DN70–DN77: Inflammatory diseases of female pelvic organs	9.76 (8.50; 11.21)
DN80–DN98: Noninflammatory disorders of female genital tract	11.82 (11.27; 12.39)
**XVIII: Symptoms, signs, and abnormal clinical and laboratory findings, not elsewhere classified (DR00–DR99)**	
DR00–DR09: Symptoms and signs involving the circulatory and respiratory systems	1.07 (0.90; 1.27)
DR10–DR19: Symptoms and signs involving the digestive system and abdomen	9.22 (8.71; 9.78)
DR20–DR23: Symptoms and signs involving the skin and subcutaneous tissue	2.18 (1.59; 2.99)
DR25–DR29: Symptoms and signs involving the nervous and musculoskeletal systems	0.75 (0.53; 1.07)
DR30–DR39: Symptoms and signs involving the urinary system	2.53 (2.08; 3.07)
DR40–DR46: Symptoms and signs involving cognition, perception, emotional state and behavior	1.10 (0.77; 1.58)
DR47–DR49: Symptoms and signs involving speech and voice	0.34 (0.13; 0.90)
DR50–DR69: General symptoms and signs	1.91 (1.72; 2.12)
DR70–DR79: Abnormal findings on examination of blood, without diagnosis	0.52 (0.22; 1.20)
DR80–DR82: Abnormal findings on examination of urine, without diagnosis	1.69 (0.10; 27.99)
DR83–DR89: Abnormal findings on examination of other body fluids, substances, and tissues, without diagnosis	1.36 (0.96; 1.92)
DR90–R94: Abnormal findings on diagnostic imaging and in function studies, without diagnosis	1.40 (0.96; 2.04)

The index date for a case and corresponding age-matched controls is defined as the date of diagnosis of endometriosis of the case.[1,2]
OR, odds ratio; CI, confidence interval.

1 Adjusted for age (matching variable), the region of residence, educational level, household type, labor market affiliations, and ethnicity.

2 There were no registered diagnoses related to the following subchapters: N40–N51: Diseases of male genital organs, N99–N99: Other disorders of the genitourinary system or R95–R99: Ill-defined and unknown causes of mortality.

© The Author(s) 2024. Published by Oxford University Press on behalf of European Society of Human Reproduction and Embryology.
This is an Open Access article distributed under the terms of the Creative Commons Attribution License (https://creativecommons.org/licenses/by/4.0/), which permits unrestricted reuse, distribution, and reproduction in any medium, provided the original work is properly cited.
Human Reproduction, 2024, 00, 1–9
https://doi.org/10.1093/humrep/deae273
Original Article
